# BEXIS2: A FAIR-aligned data management system for biodiversity, ecology and environmental data

**DOI:** 10.3897/BDJ.9.e72901

**Published:** 2021-11-05

**Authors:** Javad Chamanara, Jitendra Gaikwad, Roman Gerlach, Alsayed Algergawy, Andreas Ostrowski, Birgitta König-Ries

**Affiliations:** 1 Friedrich-Schiller-Universität Jena, Jena, Germany Friedrich-Schiller-Universität Jena Jena Germany; 2 TIB – Leibniz Information Centre for Science and Technology and University Library, Hannover, Germany TIB – Leibniz Information Centre for Science and Technology and University Library Hannover Germany; 3 German Centre for Integrative Biodiversity Research (iDiv) Halle-Jena-Leipzig, Jena, Germany German Centre for Integrative Biodiversity Research (iDiv) Halle-Jena-Leipzig Jena Germany

**Keywords:** research data management, FAIR data principles, open-source, biodiversity, ecology, environmental science, FAIR data maturity model, data lifecycle

## Abstract

**Background:**

Obtaining fit-to-use data associated with diverse aspects of biodiversity, ecology and environment is challenging since often it is fragmented, sub-optimally managed and available in heterogeneous formats. Recently, with the universal acceptance of the FAIR data principles, the requirements and standards of data publications have changed substantially. Researchers are encouraged to manage the data as per the FAIR data principles and ensure that the raw data, metadata, processed data, software, codes and associated material are securely stored and the data be made available with the completion of the research.

**New information:**

We have developed BEXIS2 as an open-source community-driven web-based research data management system to support research data management needs of mid to large-scale research projects with multiple sub-projects and up to several hundred researchers. BEXIS2 is a modular and extensible system providing a range of functions to realise the complete data lifecycle from data structure design to data collection, data discovery, dissemination, integration, quality assurance and research planning. It is an extensible and customisable system that allows for the development of new functions and customisation of its various components from database schemas to the user interface layout, elements and look and feel.

During the development of BEXIS2, we aimed to incorporate key aspects of what is encoded in FAIR data principles. To investigate the extent to which BEXIS2 conforms to these principles, we conducted the self-assessment using the FAIR indicators, definitions and criteria provided in the FAIR Data Maturity Model. Even though the FAIR data maturity model is developed initially to judge the conformance of datasets, the self-assessment results indicated that BEXIS2 remarkably conforms and supports FAIR indicators. BEXIS2 strongly conforms to the indicators Findability and Accessibility. The indicator Interoperability is moderately supported as of now; however, for many of the lesssupported facets, we have concrete plans for improvement. Reusability (as defined by the FAIR data principles) is partially achieved.

This paper also illustrates community deployment examples of the BEXIS2 instances as success stories to exemplify its capacity to meet the biodiversity and ecological data management needs of differently sized projects and serve as an organisational research data management system.

## Introduction

The authors of the Summary for Policymakers of the Global Assessment Report on Biodiversity and Ecosystem Services of the Intergovernmental Science-Policy Platform on Biodiversity and Ecosystem Services (IPBES) identified knowledge gaps and indicated access to more multidisciplinary biodiversity data and information as crucial needs (Díaz et al. 2019). However, obtaining fit-to-use data associated with diverse aspects of biodiversity is difficult since often it is fragmented, sub-optimally managed and available in heterogeneous formats.

Conventionally, in the biodiversity, ecology and environmental sciences domain, data used to address research questions are embedded inextricably in scientific publications as the basis for research findings in the form of tables, graphs and supplements. To promote data availability and reproducibility of results, there is a growing demand from journal publishers, funders and government agencies, that the authors publish the underlying dataset along with associated metadata in scientific data repositories or data journal (Deutsche Forschungsgemeinschaft (DFG) 2013, Deutsche Forschungsgemeinschaft (DFG) 2015, Directorate-General for Research and Innovation (European Commission) 2018, Marcial and Hemminger 2010, Penev et al. 2017, Roche 2017, Science Digital et al. 2018, COPDESS 2014, Springer Nature 2021, Chavan and Ingwersen 2009, Roberts and Moritz 2011). Recently, with the universal acceptance of the FAIR (Findable, Accessible, Interoperable, Reuseable) data principles (Wilkinson et al. 2016), the requirements and standards of data publications have changed substantially as compared to former times. To improve the findability, accessibility, interoperability and reusability of research data, various stakeholders across the globe (Directorate-General for Research and Innovation (European Commission) 2018, Wittenburg et al. 2020, Sales et al. 2020, Dutch Research Council (NWO) 2019, Science Digital et al. 2018)have adopted the FAIR data principles as requirements to facilitate reproducibility, transparency and discovery of knowledge. Researchers are encouraged to manage the data appropriately and ensure that the raw data, enriched metadata, processed data, software, codes and associated material are securely stored and in accordance with the FAIR data principles and the data be made available with the completion of the research. While the FAIR data principles do not explicitly address (meta-)data quality, employing them contributes to high data quality. Recent suggestions to extend the notion of FAIRness to software (Lamprecht et al. 2020), study designs and workflows (Harjes et al. 2020) further strengthen this aspect.

In recent times, best practices based research data management is actively considered during the course of the research in the field of biodiversity, ecology and environmental sciences (Deutsche Forschungsgemeinschaft (DFG) 2013, Deutsche Forschungsgemeinschaft (DFG) 2015). However, there exists a gap between the expectations of different stakeholders and the ability of the researchers to implement in reality due to inadequate research data management skills, paucity of supporting infrastructure and tools and limited financial resources (Miyakawa 2020, Baker 2016, Assante et al. 2016, Stuart et al. 2018, Smits and Teperek 2020, Mons 2020, Alves et al. 2018).

As observed by Mons 2020 and Stuart et al. 2018, in general, researchers and research organisations are overwhelmed by the challenges of managing data appropriately due to the time and efforts involved. Research data management is a fundamental component of the research lifecycle and it typically starts from the conceptualisation of a project and accompanies it to its finish. It is a process in which the data go through different conceptual stages such as collection, analysis and publication (Recknagel and Michener 2018).

In order to meet the objective of making biodiversity and ecology data openly and easily available using best research data management practices, in this paper, we describe the open-source community-driven development of a web-based data management system BEXIS2 (http://bexis2.uni-jena.de/). The BEXIS2 system is designed to provide support for the entire data lifecycle throughout the active project phase where researchers collect, document, process, integrate, analyse and share data. When designing BEXIS2, we took great care to ensure its adaptability to new challenges and paradigms via a modular architecture.

In the ensuing sections, we outline how the system works, its architecture, salient features and interoperability with other data platforms. Further, as a novel approach, we describe the outcomes of the BEXIS2 conformity assessments with the FAIR data principles using the Research Data Alliance FAIR Data Maturity Model (Working Group, Research Data Alliance FAIR Data Maturity Model 2020). Finally, through examples, we exemplify BEXIS2 capacity to meet the biodiversity and ecological data management needs of different projects, as well as to serve as an organisational research data management system.

## Project description

### Title

BEXIS2 Research Data Management System

### Study area description

Research data management for biodiversity, ecology and environmental data

**1. System Description and Utility**

BEXIS2 is a web-based modular and extensible system (https://youtu.be/sLTAmQLTSBA) providing a range of functions from data structure design to data collection, data discovery, dissemination, integration, quality assurance and research planning. The system provides a collaborative research data management platform to support various stakeholders, such as researchers, data managers, citizen scientists and project coordinators to manage and share their data mainly during the projects’ lifetime and beyond.

BEXIS2 is released in source and binary formats; however, there is no central organisation/authority to run the application. Any project or organisation that is interested in the system’s functionality may deploy it on its own infrastructure and make it available to its audience. Each running deployment of the application is called an “instance”.

**Figure 1.**
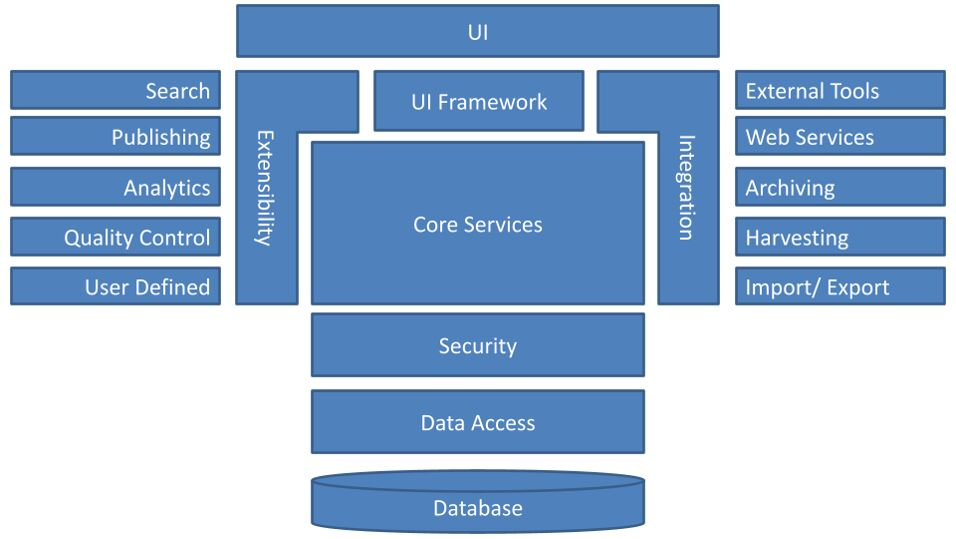
The overall BEXIS2 architecture. The modules on the extensibility side are exemplary; the implemented modules may differ.

The overall architecture of the system is shown in Fig. 1 and explained in detail at https://youtu.be/txLIDgEn59M. The Core Functions component manages the data lifecycle. All the functions pass through a Security layer to be able to access the data. The core functions are accessible through: (1) the User Interface for human consumption, (2) theIntegration layer for the machine to machine communication and integration with external systems and (3) the Modularity layer for extensibility purposes.

BEXIS2 is developed in the C# language and has adopted a modified Model-View-Controller (MVC) pattern. It utilises ASP.NET (Version 4.5.2) MVC and deploys it onto MS IIS. The current version of BEXIS2, which was initiated in the year 2011, initially relied on the code developed for the Biodiversity Exploratories Information System (BExIS) in 2007 (Lotz et al. 2012), as well as some open-source contributions, especially for multi-tenancy and multi-RDBMS support. The use of MVC was an architectural decision to separate the application logic, presentation logic and the actual User Interface from each other. This improved testability of the whole software and allowed the team to quickly wrap the application logic into REST APIs for integration with external tools and programming languages. MVC patterns with REST API support, routing, route versioning and route-based access control mechanisms were more practical and robust in .NET at the time of design, compared to Python, for example. BEXIS2 uses NHibernate as Object-Relational Mapping (ORM) to build an abstract layer between the application logic and the underlying database management systems (DBMS). BEXI2 is tested on and released with PostgreSQL as the default relational DBMS.

The Core Functions component of the system relies on a conceptual model (Chamanara and König-Ries 2014,https://youtu.be/tqjJ56Pjp0I) that defines and formalises the main concepts on which the software is built. The cornerstone of the conceptual model as illustrated in Chamanara 2012 is the term “**dataset** ”.

**Figure 2.**
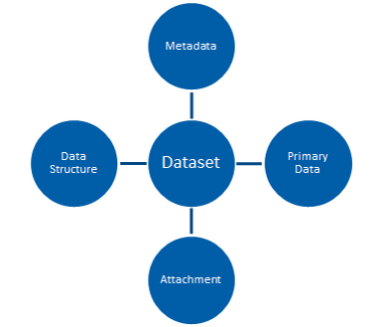
The main elements of a dataset in the BEXIS2 system.

Within the context of the BEXIS2 system, as illustrated in Fig. 2, a dataset is a compound that consists of four main elements, which are: (1) **Primary data**, (2) **Data structure**, (3) **Metadata** and (4) **Attachments** (*Chamanara and König-Ries 2014*).

**Primary data**

The primary data are a container for structured (tabular) and unstructured data, such as observations, measurements, simulations and multimedia. A tabular dataset is a specific type of structured data that consist of a set of tuples (rows), where each tuple is a collection of cells containing the actual data (Chamanara et al. 2015). Each cell represents a value resulting from observations, measurements, computations, simulations or any other means of data acquisition.

**Data Structure**

To define how the data are organised within a dataset, a data structure is used. For tabular data, the data structure can be specified by data types (e.g. Numeric, String, DateTime), units of the measurements, description, encoding of missing values and constraints (e.g. valid data range, valid terms) associated with the variables. A data structure refers to one single table. Each dataset can have only one data structure. Different datasets and research projects have various study purposes, causing high diversity of the structures of the primary data (Chamanara et al. 2015).

BEXIS2 provides functionality to define these structures for individuals or groups of datasets either in advance or during the data submission. In addition, the reusable elements, such as the units of measurements, unit conversion information, data types and data validation rules, are factored out to potentially enable data sharing, integration and cross dataset semantic querying.

**Metadata**

The metadata elements capture information associated with the dataset, such as the owner(s) of data, project(s), involved researchers, spatio-temporal extent of the work and access, sharing and publishing policies. In contrast to many other systems, in BEXIS2, metadata can vary by project, institution or publication purpose. Instead of conforming to any specific metadata and/or data standards, such as the Ecological Metadata Language (EML) (Jones et al. 2019), Darwin Core (Wieczorek et al. 2012), Dublin Core or Access to Biological Collection Data (ABCD) (Access to Biological Collection Data Task Group 2007), BEXIS2 provides a mechanism that allows authorised users to design or import metadata schemas that fit their requirements.

**Attachments**

The attachments are auxiliary files associated with a dataset. They can be experiment designs, laboratory notes, drafts, analysis programmes and scripts, visualisations, images, videos, sounds, user manuals or any other supporting material.

Datasets may be edited over time for different reasons, for example, cleaning, review and incremental completion. At the same time, it is possible that datasets are used in computations, publications or otherwise cited. BEXIS2 has incorporated a linear versioning scheme with checkout (the operation that creates a new version of the dataset and makes it editable), check-in (the operation that applies all the changes made on the dataset from the checkout time onwards) and discard (the operation that discards all the changes made to the dataset from the last checkout, removes the open editing version and takes the dataset to the latest checked-in version) operations on datasets that keeps all the previous versions unaffected by the changes introduced. This enables the dataset publishers to publish a version while working on the next version of a dataset. It is also possible to have multiple versions of a dataset published independently, even by different publishers. Each version of a dataset obtains a unique local identifier and a global identifier when published. This allows unambiguous retrieval by analysis programmes or citations in scientific articles.

From the usage perspective of a data management system, such as the BEXIS2, it involves at least three key different roles which are: (1) **Researchers** (2) **Data Manager** and (3) **Developers**. The following subsections describe concepts, design decisions and system features relevant to these roles.

**Researchers**

Researchers in academia are the primary group of users of the BEXIS2 system. A researcher creates data that need to be securely stored and prepared for later reuse by peers or other interested parties. These users are able to ingest data, describe the data with metadata and publish them by different means. They are automatically informed about events that have happened to their data during its lifecycle. For example, they obtain emails when someone downloads their dataset. A request system exists. With this, researchers are informed, if someone requests access to their dataset and can grant or deny that request.

Researchers may submit their data at any stage of their research. They can update the data and the metadata and rely on the versioning capability of the system to keep track of the changes and allow them to go back to a previous version if needed. They can also retrieve any version of the dataset at any time. Following the principles of good scientific practice, BEXIS2 does not allow the deletion of data by researchers.

BEXIS2 is a collaborative data management platform where a conscious choice was made not to provide domain/community-specific data analysis features within the system. Rather, the system does provide the means to access data with external tools, for example, R, so that it can be easily integrated into a researcher's workflow.

Besides the data producers' perspective, researchers also retrieve data from BEXIS2 for further reuse. Thus, BEXIS2 provides facilities for researchers to search and discover datasets, uniquely identify them, access the datasets either manually or via software systems and programming languages and explore the content of the datasets including their metadata, schema and supporting materials.

Searching amongst the metadata, data structure and primary data in an integrated way is an intrinsic feature of the system. For example, variable names or measurement units can be used as a keyword to search datasets. Search results provide access to full metadata and the public components of the data, for example, version history, the status of the data and publishing information. Based on the access rights, users may be able to browse and download the primary data, too.

The system allows for the discovery of data, based on metadata attributes, via, for example, facets, properties and scopes, either individually or in combination with free search text provided by the users.

The dataset can be accessed manually via the web interface as well as using computer programs, such as Python, Java, R. Metadata, primary data and their structures can be created, updated and retrieved through the user interface (GUI), as well as the system APIs over protocols, such as HTTP or OAI-PMH in HTML or XML serialisation formats. For example, depending on the user access permissions, these APIs allow researchers to obtain the designated dataset using their programming language of choice and integrate the data retrieval into their data processing and analysis pipelines. It is also possible to retrieve a specific version of the chosen dataset to isolate the analysis programme and its results from the potential future changes that may apply to a designated dataset. The BEXIS2 API delivers the primary data, along with the data structure.

**Data Managers**

Within the BEXIS2 context, a data manager is responsible for supporting researchers in using the system, ensuring data quality and system integrity. Data managers may also support developers by providing feedback on user experiences and specifying requirements to improve the system. For this paper, we do not distinguish between different supporting roles like data manager or curator, but subsume them under data manager.

BEXIS2 is released with an extensible collection of biodiversity domain-specific metadata schemas, based on ABCD and EML. They may, in addition, choose to reuse or customise existing metadata schemas towards the needs of their community. Data managers may also design metadata schemas that best fit their requirements. Additionally, BEXIS2 utilises a comprehensive multi-metadata schema search mechanism, where data managers have full control of specifying metadata attributes and mapping them to facets, categories or properties used in the search. This way, the data managers can shape what is searchable and how those searchable items are shown to the users on the user interface. Furthermore, BEXIS2 provides the possibility to search not only using metadata attributes, but also primary data.

Data managers can support the design of custom data structures for the projects and individual datasets in their domain. They can do so by specifying sets of variables, units of measurements and data types. At present, annotating the units and variable properties to appropriate ontologies is under implementation.

Further, data managers can set permissions to control which individuals and groups are authorised to create, view (including download) or delete datasets. Additionally, they can determine who can grant data access permissions. Besides data permissions, data managers are also able to specify permissions on system features (i.e. functions).

Data Managers can configure the user authentication mechanism. In addition to the stand-alone mechanism implemented in BEXIS2, it is also possible for them to integrate with their organisational identity management system, such as the Lightweight Directory Access Protocol (LDAP) and Active Directory.

If the BEXIS2 instance hosting organisation is authorised, the data managers can register the BEXIS2-generated unique identifiers with persistent identifier granting authority using the additional plugin. These identifiers resolve to the landing page of the corresponding datasets. A landing page provides access to all of the elements of the designated dataset including metadata, primary data, data structure, supplementary material and published versions. In addition, data managers can potentially publish datasets (metadata and/or data) to external platforms such as the Pensoft Biodiversity Data Journal and German Federation for Biological Data (GFBio) and assign permanent identifiers (e.g. DOI assignment via DataCite registry) for published versions of datasets. Metadata (and data) formatting/packaging required by external repositories, as well as licensing information, can be configured in the system. Facilities for data quality control by means of peer reviews and helpdesks are under consideration for future development.

The system also provides facilities for data managers enabling them to define configurations that apply to the entire instance as well as the project or organisation-specific rules. This covers legal terms and conditions, licences and even the appearance of the user interface. These higher-level policy enforcement mechanisms do not constrain individual datasets from having their own licence.

Besides, data managers can also perform all actions that regular users can perform including creating new datasets, as well as editing the elements of existing datasets such as metadata, primary data and supplementary materials.

**Developers**

BEXIS2 is an extensible and customisable system. It allows for the development of new functions and customisation of its various components from database schemas, to the user interface layout, elements and the look and feel. Additionally, as open-source software that is developed and maintained on BEXIS2 GitHub Repository, it always welcomes and appreciates contributions from the community.

As depicted in Fig. 1, the system is designed with a highly modular and decoupled architecture from the user interface to the core of its domain and conceptual model. This opens up the doors for enhancements, innovations and customisation towards the specific needs of different communities and scientific domains.

There are various ways the developers can contribute to the system, such as:

**System Enhancement**: If a developer wants to refactor a piece of code, enhance some functions/elements or fix an issue, the developer can clone the source code from the project’s GitHub repository and apply the changes. The developer has the option to contribute to the main codebase by creating a pull request and asking the project’s core team to merge it. Although not limited to, this type of enhancement may add new features to the user interface, design and apply new themes and visual elements.

**Module Development**: A module is a package of related functions that are developed, tested, deployed and used together. The default functions of the system are also developed using this approach. The module development has extensive documentation (https://fusion.cs.uni-jena.de/bpp/bexis-2-tech-talk-series/), as well as boilerplate code (https://github.com/BEXIS2/ModuleTemplate), to reduce the module development initiation and development efforts. Modularisation opens up many possibilities to replace existing functionalities, but in particular, to add own functionalities. Developers can easily expand the system and adapt it to their own needs. For example, the Central Data Management Project of the Biodiversity Exploratories provides a set of modules that are also maintained on GitHub (https://github.com/bexis) and are freely available to fulfil specific needs of projects, such as fieldwork organisation, index calculation, resource planning, publication management and event management. For details, see the section Biodiversity Exploratories Information System under Typical BEXIS2 Deployments.

**Functionality Extension**: Many elements of the system are designed with extension in mind, namely, they provide extension points with well-defined interfaces. For example, at the data access level, there are extension points that fire events before and after transactions are committed/rollbacked. Each module provides a REST API to its internal functions. In addition, the modules provide user interface extension points and configuration items that control their appearance and placement on their upper layer element. For example, the data collection module has a scenario to submit a dataset to the system, which is realised using a workflow. The workflows are also customisable, therefore, a developer is able to change the scenario and adapt it to the requirements of its data managers and researchers. The modular design of the system allows for easy integration with external systems, for example, assigning DOI minting to the published datasets. A developer, familiar with REST API design, can also combine various module level APIs to build up higher-level functions.

**System Integration**: BEXIS2’s well-defined APIs can be easily exploited by consuming them from external systems. This enables organisations to integrate BEXIS2 services with their organisation-wide software ecosystem. For example, authentication of the user can be integrated into the organisation's user base. As another example, in BEXIS2, metadata and its associated structure are offered via the OAI-PMH standard. External harvesters and search engines can obtain the metadata and index it for their own purposes, namely, to combine it with their community-specific data and serve their audience.

**2. FAIR Data Principles Conformance Assessment**

When we started to build BEXIS2, the FAIR data principles did not yet exist. Nevertheless, we aimed to incorporate key aspects of what is encoded in those principles into BEXIS2 from the very start. For instance, it was always our aim to make it easy to find and reuse data. In this section, we investigate to what extent BEXIS2 meets the FAIR data principles. One problem in this endeavour is that the FAIR data principles themselves are described rather generically and often leave room for interpretation. We, therefore, decided to follow the more precise definitions and criteria provided in the FAIR Data Maturity Model published by the respective Research Data Alliance (RDA) working group (Working Group, Research Data Alliance FAIR Data Maturity Model 2020). For each of the FAIR data principles, it provides a number of indicators, estimates their importance and gives hints on how to evaluate them. For the evaluation, two different perspectives can be taken, a simple “pass-or-fail” view or a view on the progress made towards achieving this goal. Given the fact that BEXIS2 is under active development and continuously evolves, we opted for the latter option.

In the following, we first provide a brief description of our approach and then the detailed results of the self-assessment structured along the four FAIR data principles.

**Self-assessment**

Different methods have been introduced overtime for the assessment and self-assessment of data and software (Bahim et al. 2019, Bahim et al. 2020). In this section, we iterate over the RDA FAIR indicators and explain how BEXIS2 realises them. These indicators have originally been developed to judge the conformance of datasets to the FAIR data principles. In this paper, however, we apply them to the BEXIS2 software system and conduct a self-assessment to grade BEXIS2’s conformance to the indicators. Tables 1-4 summarise the conformance of BEXIS2 to each of the FAIR data principles. The **FAIR Indicator** column is the RDA indicator. The letter in brackets indicates its importance (Essential, Important or Useful) assigned to this indicator by the RDA. The **BEXIS2 Feature** column is a short explanation that shows how BEXIS2 realises the indicator and the **Score** column is our self-assessed value (in the range of 0-4 as defined by the RDA maturity model) that shows the extent of conformance of BEXIS2 to the corresponding indicator. The score was determined by independent assessment of BEXIS2 conformance by five of the co-authors of this paper and subsequent discussions, which, in some cases, resulted in a re-evaluation. The final score is the rounded average of those provided by individual assessors. In the column **Availability**, we also indicate whether the system guarantees that the indicator is met or whether it enables meeting them, but the level of fulfilment of a specific BEXIS2 instance depends on the decisions of the data manager of that instance. For example, BEXIS2, by design, provides unique identifiers, so conformance to F1-02M is guaranteed. On the other hand, BEXIS2 allows data managers to choose which metadata schema to use. With this, it enables the fulfilment of I1-01M, but cannot guarantee it. Here, ultimately, meeting the requirements is the responsibility of the data manager. The fact that BEXIS2 data managers can configure their instance of the software quite freely prevents us from using one of the existing FAIR assessment tools like DataONE 2021 and Wilkinson et al. 2019, to assess the FAIRness of the software as such. It is recommended though for data managers to use these tools to evaluate their configurations.

**Findability (F)**

If one wants to use data, one needs to first be able to find it. In order to do this, in the “Findability” part of the FAIR data principles, features that enable humans and machines to discover datasets are identified. In the following Table 1, we look at each of those and evaluate how well BEXIS2 meets them.

In summary, BEXIS2 strongly supports all aspects of “Findability” by providing unique, persistent identifiers, direct integration of the DOI mechanism and OAI-PMH based metadata export and harvesting facilities.

**Accessibility (A)**

The “A” in FAIR stands for Accessibility, i.e. whether a data source provides sufficient information on how data and metadata can be accessed technically, for example, whether/which authentication is needed or which community-relevant schemas, standards and access protocols are used and how open these protocols are (Table 2).

BEXIS2 offers data and metadata access both via a GUI and an API and using a variety of standardised protocols and formats. Thus, overall, accessibility is reached to a high degree. Most indicators are guaranteed by the system, so every BEXIS2 instance will fulfil them.

**Interoperability (I)**

Most often, data are not used in isolation, but need to be integrated with other data and ingested by tools and workflows. The “I” indicators assess how easy that is (Table 3).

As of now, BEXIS2 does not fully meet the interoperability indicators. However, for most aspects, concrete improvements are planned or already under implementation. Even with these improvements, by their very nature, fulfilment of most indicators in this section will be the responsibility of the data managers- BEXIS2 will be better able to support them once the ongoing implementations are completed, though.

**Reusability (R)**

The “R” indicators collect a number of aspects related to how easy it is to reuse data. They cover a wide range of topics from the quality of the description, to clear licences and provenance information (Table 4).

As with interoperability, BEXIS2 still needs extensions to really meet the "R" requirements. As with the “I”, many of the “R” indicators fall within the responsibility of the data managers.

**Assessment Summary**

**Figure 3.**
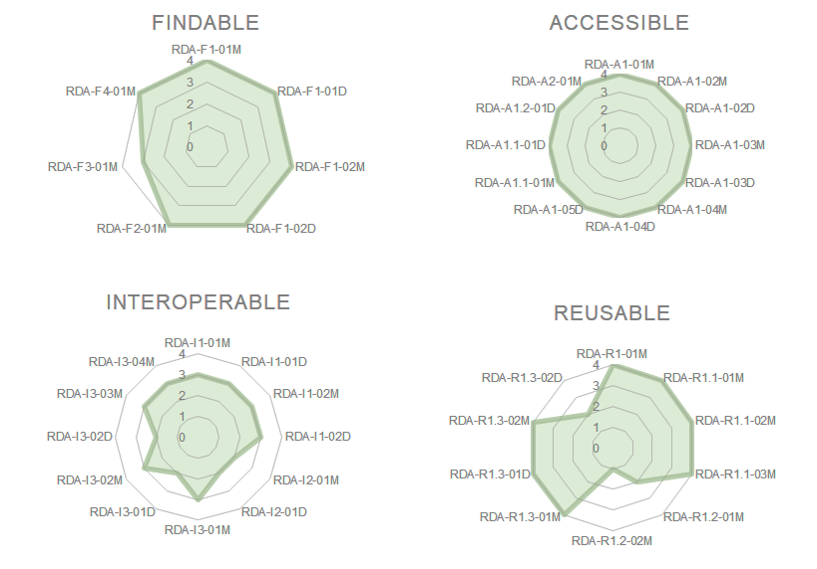
Spider net (Radar) charts illustrate BEXIS2 conformance to the RDA FAIR data maturity model. **Maturity level per indicator (per FAIR area)** - 0 – not applicable; 1 – not being considered as yet; 2 – under consideration or in the planning phase; 3 – in implementation phase; 4 – fully implemented.

In Fig. 3, we have summarised the findings from the Tables provided above. The Spidernet charts visualise the level of compliance with the individual FAIR criteria. The diagram indicates that BEXIS2 is already very mature with respect to Findability and Accessibility. Interoperability is moderately supported as of now; however, for many of the less-supported facets, we have concrete plans for improvement. Reusability (as defined by the FAIR data principles) is partially achieved. In particular, for various domain and study-specific digital objects, the use of community standards for provenance information is still lacking.

It is important to note, for data managers, that some of the indicators will, by default, be met by any BEXIS2 instance. This is true, for instance, for all of the accessibility indicators. For others, the system supports or at least allows settings that will lead to meeting the indicators, but choosing the right settings and providing the right infrastructure ultimately is the responsibility of the individual instance. Data managers have to be aware of this responsibility. For example, BEXIS2 supports workflow for minting DOIs; using this functionality, however, requires the existence of a contract of the BEXIS2 instance hosting organisation with DataCite. Another example is the usage of standards for data and metadata - this is possible, but not enforced by the system.

**3. Typical BEXIS2 Deployments**

In this section, we highlight some usage scenarios of BEXIS2 by describing a variety of typical deployments of the software. These examples show how the configurations and customisations described above can be used to tailor the system to a specific project’s needs. As part of active community contribution towards the core development of the BEXIS2 system, we also introduce examples of modules that were developed to cater for specific projects, but are now available to serve the wider community.

**iDiv Data Repository**

The German Centre for Integrative Biodiversity Research (iDiv) Halle-Jena-Leipzig is a large-scale collaborative biodiversity science effort that promotes theory-driven synthesis and data-driven theory. There are more than 200 scientists directly employed by iDiv and several hundred associated with it. They work in five main research areas of biodiversity science: complexity, functioning, change, molecular and society. The researchers produce and use complex datasets that belong to multidisciplinary topics including ecometabolomics, traits, plot inventories, species distribution, taxonomy, land-use, sensors and environmental with spatial and temporal dimensions ranging from molecule to global. The data sources vary from field collections to data aggregated from various online data platforms, such as the Global Biodiversity Information Facility (GBIF), Biodiversity-Ecosystem Functioning Project in China (BEF-China), Biodiversity Exploratories and TRY Plant Trait Database.

**Figure 4.**
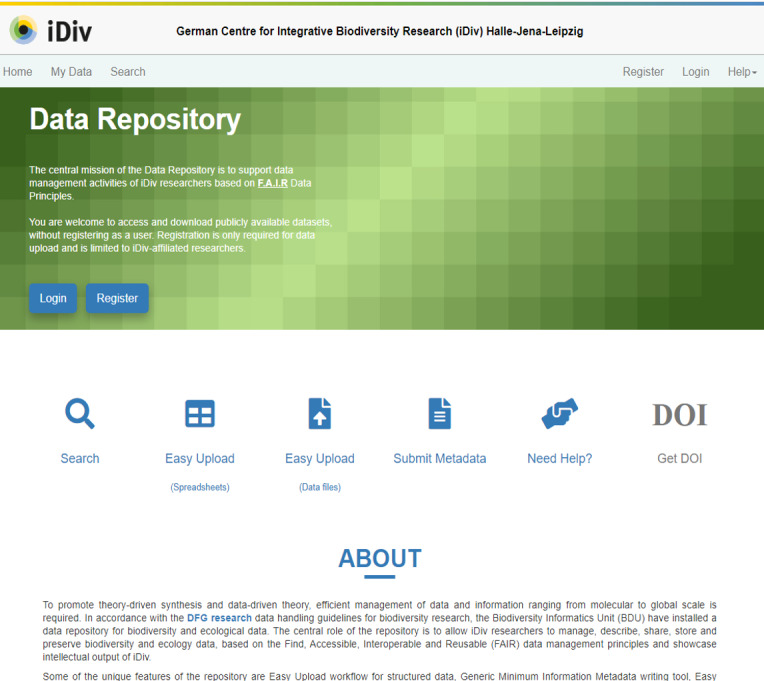
iDiv data repository home page.

**Figure 5.**
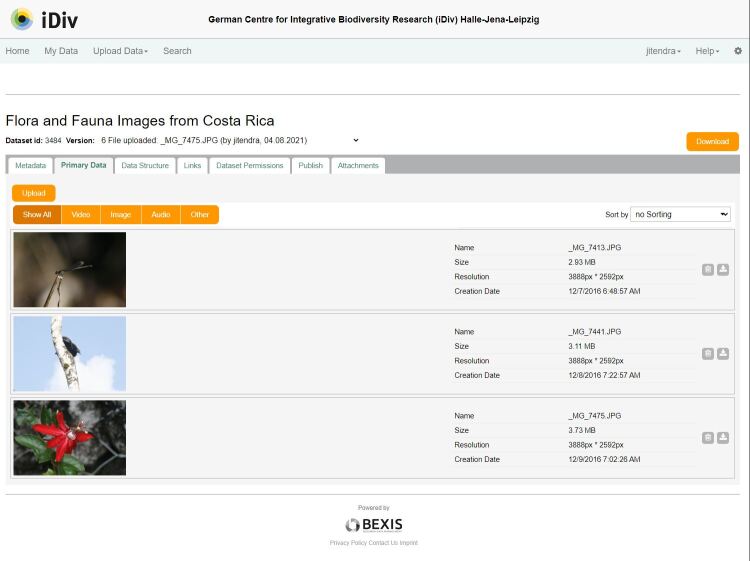
Multimedia module developed by iDiv integrated as part of the core BEXIS2 system.

For efficiently managing diverse biodiversity data coming from different resources in heterogeneous formats, there was a need for an easy-to-use tool that could support the data management activities of a large number of rather loosely cooperating researchers. The central role of the iDiv data repository (https://idata.idiv.de) is to allow iDiv researchers to manage, describe, share, store and preserve biodiversity and ecology data, based on the Find, Accessible, Interoperable and Reusable (FAIR) data principles and showcase the intellectual output of it (Fig. 4). Some of the unique features of BEXIS2 for the repository are the easy upload workflow for importing structured data, metadata writing tool (RDA-F2-01M; RDA-R1.1-01M), easy mapping of variables with controlled vocabularies (RDA-I1-01D; RDA-I2-01D) and data standards, such as Dublin Core (DC), Darwin Core (DwC) and Ecological Metadata Language (EML) (RDA-I1-01M; RDA-I1-02M). The modular features of the BEXIS2 provided the flexibility to customise and extend (Fig. 4) as per the data management requirements of different user groups in iDiv, such as the development of the Multimedia module (Fig. 5).

Many research projects in iDiv are using high definition audio-video-images capturing tools, such as high-resolution cameras, drones, acoustic recorders and sensors for conducting long-term ecological and biodiversity studies. However, the ability to efficiently manage and effectively retrieve multimedia data was lacking in the BEXIS2 system. The generated multimedia data were stored in file systems and accessing required familiarity with the data and the storage structure. This significantly hampered, in particular, the reuse of multimedia data and slowed scientific progress. As part of the BMBF-funded project MAMUDS (Managing Multimedia Data for Science), iDiv developed a multimedia data management and content-based retrieval system geared towards biodiversity science, which was integrated as a module in the BEXIS2 application (Fig. 5). At present, the iDiv data repository contains more than 1767 datasets (Open access and Restricted), 130 registered users, provides various metadata schemas (RDA-F2-01M; RDA-A1-01M; RDA-A1-02M; RDA-R1-01M; RDA-R1.3-02M) for thematically diverse data-intensive projects (Phenology, Long-term monitoring, Ecometabolomics, Sample Event) and assigns DOIs to the datasets (RDA-F1-01M; RDA-F1-01D; RDA-F1-02M; RDA-F1-02D; RDA-A1-02D; RDA-A1-03M; RDA-A1-03D) which is done by interacting with the DataCite application using the APIs. Apart from the data repository, iDiv is also hosting a dedicated instance of the BEXIS2 system for the data-intensive project, the Jena Experiment (https://jexis.idiv.de/), in order to support efficient data management activities. Besides the BEXIS2 core development team, iDiv actively contributes codes, bugs fixes, feature development, such as the data structure creation and documentation for the BEXIS2 application.

**AquaDiva Data Portal**

The Collaborative Research Centre (CRC) AquaDiva is a large collaborative project spanning a variety of domains, such as biology, geology, chemistry and computer science. The common goal of the CRC is to better understand the Earth’s critical zone, in particular, how environmental conditions and surface properties shape the structure, properties and functions of the subsurface. Within AquaDiva, large volumes of heterogeneous observational data are being collected. Besides this structured data, knowledge is also encoded in an unstructured form in scientific publications. A first step to automate the integration, discovery and reuse of these datasets is to allow scientists from different groups to upload/update their datasets. To achieve this task, we make use of the BEXIS2 system as the infrastructure to organise and store data. The CRC AquaDiva is organised into four main groups: (1) BIODIV- biodiversity and ecosystem functioning, (2) FLUX- fluxes and elements flow, (3) GEOFACT- interactions at subsurfaces solid/fluid interface and (4) INFRA- infrastructure and data management. The total number of registered users with the AquaDiva Data Portal (ADDP) (https://addp.uni-jena.de/) is more than 150 users distributed in different roles, as data managers, developers and researchers. Each group has several subgroups, each producing different, but related datasets. This demonstrates the capability of BEXIS2 that supports AquaDiva to deal with different data resources, such as weather monitoring, groundwater hydrochemistry, soil physical parameters etc. As a result, we consider BEXIS2 as the core infrastructure for AquaDiva Ontology-based Information System (ADOnIS) (Algergawy et al. 2018). Each data file (structured and/or unstructured) is managed as a separate dataset with its own metadata and a data structure, if necessary. While independent, these datasets are not isolated: BEXIS2 provides the possibility to share and link at different granularities, such as data attributes and data structures. For example, even though more than 250 datasets have been uploaded to the AquaDiva Data Portal, only 150 different data structures are used. This means that the same data structure can be re-used by different datasets.

In the following, we demonstrate how BEXIS2 is used to support the implementation of FAIR data principles within the CRC AquaDiva. To support the findability of a dataset (either metadata or primary data), BEXIS2 provides the possibility to assign a unique identifier to each dataset within the same workspace. This can be achieved by assigning a dataset identifier, as well as the constraint that there are no two datasets with the same title (RDA-F1-01M/01D). Furthermore, BEXIS2 affords to make use of one of the predefined standard metadata or import project-specific metadata (RDA-F2-01M). In the CRC AquaDiva, we make use of the ABCD and EML metadata standard, as well as the AquaDiva-specific metadata. Finally, BEXIS2 also has a service that allows the permitted users to gather and export required metadata (RDA-F4-01M).

**Figure 6.**
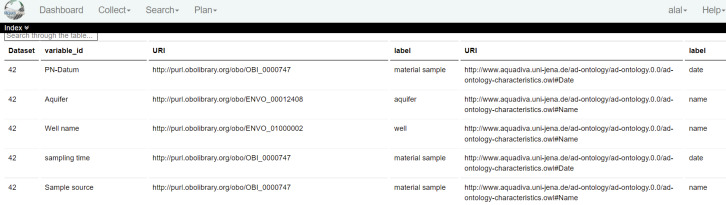
Use of AD ontology to annotate datasets.

**Figure 7.**
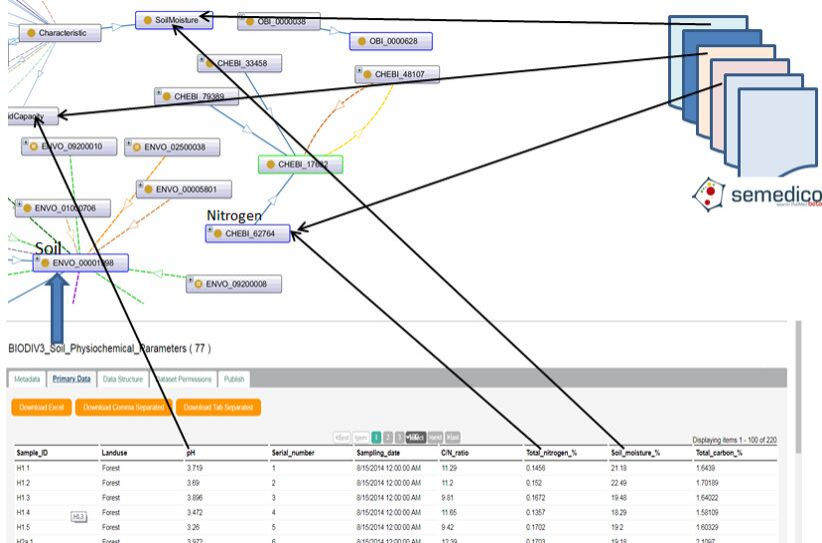
Using AD ontology (ADOn) to link between different datasets.

BEXIS2 provides the possibility to access different pieces of datasets by allowing the search through metadata and/or primary data. Exploiting features provided by BEXIS2, ADDP includes as much information as needed in metadata to enable the user to get access to the data (RDA-A1-01M), which can be achieved manually (RDA-A1-02M) or automatically making use of standard protocols (RDA-A1-04M). Similarly, it allows access to primary data, either manually with the human intervention (RDA-A1-02D) or automatically using the user interface (RDD-A1-05D) by making use of standard protocols (RDA-A1-04D). Exploiting the modularity feature of BEXIS2, we extend it to allow free access to metadata for all members of the CRC AquaDiva (RDA-A1.1001M), while only authorised members have access to primary data (RDA-A1.2-02M). Due to the modular nature of BEXIS2, we could develop a semantic-based layer on top of it that allows the annotation of datasets (in the particular data structure of the dataset) using the AquaDiva Ontology (ADOn). By this annotation scheme, AquaDiva could ensure that a part of the dataset is represented using standard knowledge representation (RDA-I1-01M/02M, RDA-I1-01D/02D). Fig. 6 shows the dataset (with ID 42) and a number of its data attributes, where each data attribute is annotated with two pieces of information. For example, the data attribute "Well name" is annotated with the characteristic "name" for the entity "well". Furthermore, this annotation scheme preserves links between different datasets at the data attribute level, i.e. we assume that any two data attributes from two different datasets annotated with the same ontology concept represent a link between the two datasets (RDA-I3-01M, RDA-I3-01D). This can be explained as shown in Fig. 7, where ADOn is used to link between not only tabular datasets, but also tabular datasets and relevant publications provided by Semedico (Klan et al. 2017). In the current implementation, we consider the annotation of data attributes to link them to corresponding concepts in the ADOn ontology. However, it is planned to extend the annotation scheme to consider not only data attributes, but also annotation at the data level (individuals), especially starting to build domain specific knowledge graphs.

Finally, using BEXIS2, the AquaDiva data portal is able to collect and store more than 250 datasets from different groups participating in CRC AquaDiva with more than 200 Million data points. Each dataset is associated with a number of features, such as the dataset title in metadata, data attributes definitions that support reuse of it (RDA-R1-01M). Furthermore, it provides the possibility to compile both metadata and primary data with a community standard (RDA-R1.3-01M, RDA-R1.3-01D).

**Biodiversity Exploratories Information System**

**Figure 8.**
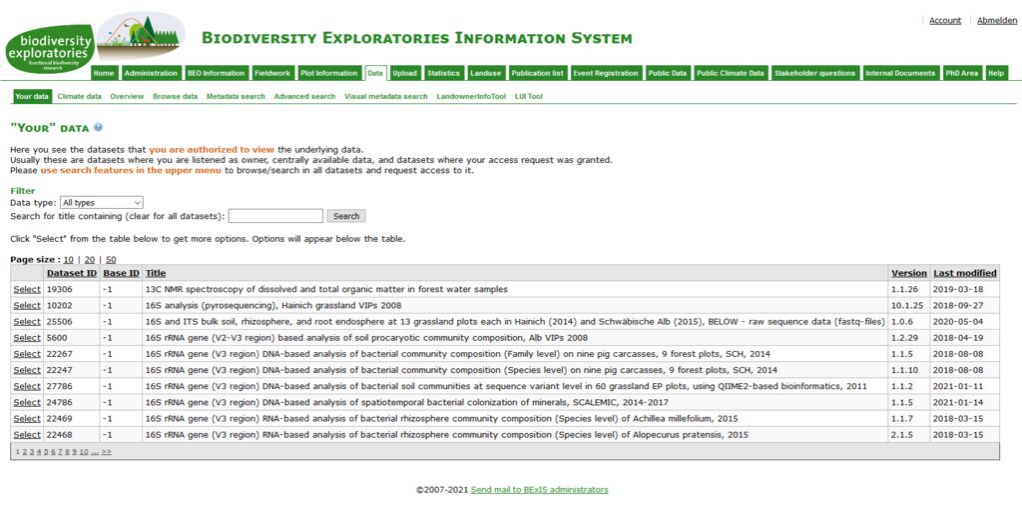
The data page of BExIS, the previous information system of the BE, showing the distributed search options on datasets in the 2nd menu level.

Established in 2006, the Exploratories for large-scale and long-term functional biodiversity research or Biodiversity Exploratories (BE) is a large-scale and long-term research project in Germany, funded by the Deutsche Forschungsgemeinschaft (DFG). The study area comprises 300 forest and grassland plots distributed across three regions in Germany (Fischer et al. 2010). Since the beginning of the BE, proper data management was considered one of the essential aspects for the success of the project. The data need to be stored safely and understandable over time. Data should be shared across all members and all phases centrally to foster collaboration. Within the BE, the online platform BExIS (Biodiversity Exploratories Information System) (Lotz et al. 2012) has been developed (Fig. 8).

BExIS provides basic functionalities common for data management system, for example, describing, structuring and uploading datasets; discoverability of datasets by use of conventional search techniques; access requesting and downloading of datasets; fundamental quality-ensuring mechanism; data versioning; and basic data analysis tools (Diepenbroek et al. 2002, Lotz et al. 2012, Michener 2015). Based on the project experience and as a result of demands from other German biodiversity domain projects, the development of BEXIS2, a more advanced and flexible research data management platform, started. The BE data management team contributed towards the development of the BEXIS2 from the beginning and the migration to the new system was agreed upon.

For many reasons, the migration to the new system took a long time. First, BEXIS2 had to provide all mandatory data management related functionalities as had been implemented in the BE. Given that the BEXIS2 development started from scratch, it took a specific development period till such a list of functions were available.

Second, within the BE project, a couple of additional non-data related features had been developed to facilitate the collaboration between people and to support the project as a whole. It provides a lot of information related to fieldwork, like interactive maps of all research plots and a booking system for field station resources and to announce plot visits. Further functionalities include a management system for publications, a document uploading mechanism, a photo gallery and an event registration tool. Most of these features were re-developed as BEXIS2 modules. For details, visit BE GitHub Repository at https://github.com/BEXIS.

Third, the already stored research data (metadata, data structure with variables and units, primary data; user and account data; rights information; data from several modules) had to be migrated to the new system. It was a great effort to make this possible. For instance, more than 20,000 user-provided variables (whereby there were more than 10,000 differently-named variables) have been reviewed and categorised into around 80 standardised variables.

**Figure 9.**
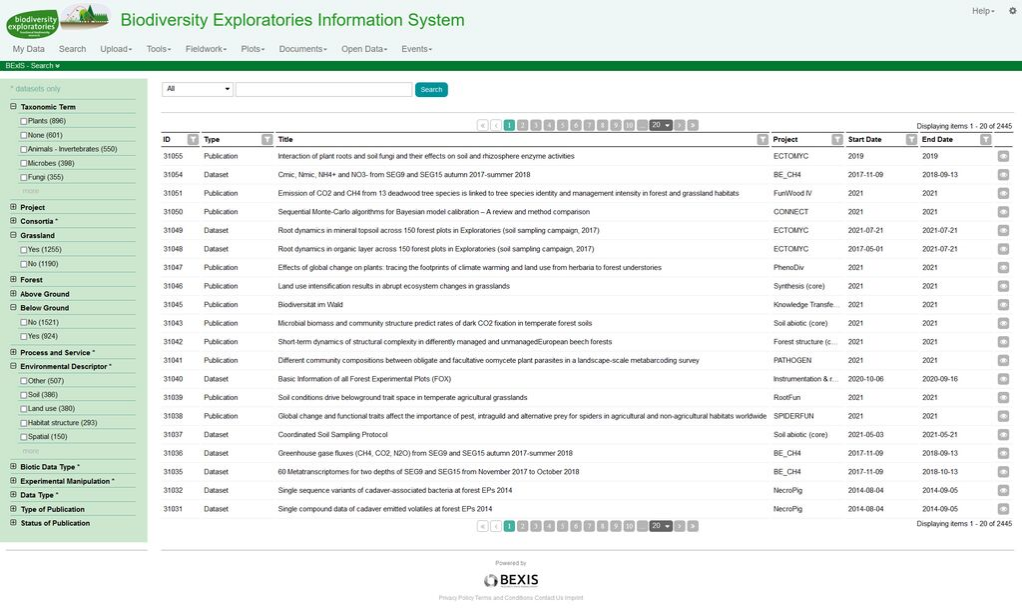
The search page of BEXIS2, the new information system of the BE, showing one integrated search amongst different entity types (here datasets and publications).

Finally, the migration was completed in February 2021. BEXIS2 is now the new software for the Information System of the BE (https://www.bexis.uni-jena.de). It contains around 1500 datasets and nearly 900 publications from more than 100 different projects since 2006 (Fig. 9).

The new system was chosen because it brings a lot of compelling data management related features and above all FAIR-tailored support for datasets within the project and to the outside. What we emphasise here are the significant improvements in relation to the previous system, such as: (i) the search interface fully integrates different entities (Fig. 9) on one page with the option to freely configure an entity shared or individual search components over their individual metadata. Such an integrated search is a dramatic boost with respect to the Findability of datasets in contrast to the previous system. This aspect is not part of the FAIR Data Maturity Model and therefore no specific indicator can be addressed here. (ii) It has an easy-to-navigate interface with a dedicated landing page for each dataset where all parts of a dataset can be accessed easily and downloaded (RDA-A1-02M, RDA-A1-02D, RDA-A1-03M, RDA-A1-04M, RDA-A1-04D, RDA-A1.1-01M, RDA-A1.1-01D, RDA-A1.2-02D), also via an API (RDA-A1-05D). (iii) By the provided option to organise the data structure via variable templates (RDA-I1-01D) easy integration of different datasets is enabled. (iv) It is possible to relate metadata fields, based on the self-chosen metadata schema, to system-wide shared attributes. This allows internally an interlinking of these metadata terms to other modules holding data (e.g. account information and needed personal data in metadata). These attributes are linked to standards, for example, Dublin Core. It enables the sharing to the outside fulfilling aspects of Findability, Interoperability and Reusability (RDA-F4-01M, RDA-I1-02M, RDA-R1.3-01M, RDA-R1.3-02M).

To sum up, the examples above show how different instances of BEXIS2 use the default features, but also the flexibility provided by BEXIS2 to tailor the system to their specific needs. This results in instances that differ in their adherence to specific aspects of the FAIR data principles.

**4. Conclusions and Future**

BEXIS2 is developed for research data management in collaborative research projects with multiple sub-projects and up to several hundred researchers. Often, these projects endeavour to address overarching questions that require sharing and integration of data across sub-projects and disciplines. BEXIS2 facilitates these data-intensive collaborations. It has been designed to support the entire data lifecycle during the active project phase where researchers collect, manipulate, document, process, analyse and share data amongst the project members. BEXIS2 provides a common platform within a project where the data are managed centrally. Like many other data platforms, BEXIS2 provides functions to ingest, search, access and download data. Besides, it provides advanced features for describing data or features supporting project management and communication. The development of BEXIS2 is based on requirements gathered from researchers of various domains, for example, biodiversity, ecology, soil sciences, biology, agriculture and atmospheric sciences. In these domains, a large portion of the data is structured as tabular data collected in the field or laboratory. In contrast to generic data management repositories (e.g. Fedora Repository, DSpace, CKAN) which mostly do not handle the internal structure of a dataset, with BEXIS2, researchers can design, store and reuse data structures and their properties. Modelling the data structure within the application facilitates data discovery and integration with other datasets. In BEXIS2, the data structures of tabular data comprise several variables, which can be defined by a data type, a unit of measurement and various other constraints. BEXIS2 encourages the reuse of (parts of) data structures across datasets. This results in similar things being modelled similarly and being easily recognised as the same thing. For example, when creating a new data structure, including, for example, a variable “temperature”, they might reuse an existing temperature variable (with a well-defined meaning). This has the potential to significantly ease cross-project data querying and integration. A detailed description of the underlying concept can be found in Chamanara and König-Ries (2014) and Chamanara et al. (2015).

As evident from the community deployment examples, BEXIS2 continues to evolve as the biodiversity and ecology community progresses towards adopting FAIR data principles and best data management practices. As the broader scientific community is growing, it is finding new avenues of usage, integrations of new modules contributed by different projects, infrastructure collaborations and services level integration with data platforms, such as the NFDI4Biodiversity, GFBio, GEO BON, GBIF and Pensoft Publications. These activities are ensuring further development and long-term sustainability of the BEXIS2 system as a product. BEXIS2 recognises that the key to ensuring this continues is to have proactive engagement with the scientific community to demonstrate the benefit of the FAIR data principles and best data management practices, foster openness and knowledge and code sharing. For instance, as part of the capacity building and community engagement, since the year 2015, BEXIS2 has been organising the BEXIS2 annual conference and hackathons. The conferences are/were well received and are participated by researchers, developers and decision-makers from different domains, such as geospatial, ecology, biodiversity, environmental, bioinformatics and computer science.

To conclude, accessing the fit-to-use biodiversity and ecological data is crucial for carrying out environmental assessments and making informed sustainable conservation policies. Researchers are encouraged to make pertinent biodiversity and ecology data available, based on the FAIR data principles for the benefit of society as a whole. It is especially imperative for megadiverse countries where the rich biological diversity is declining at an unprecedented rate. Many megadiverse countries are recognised as emerging economies exerting high pressure on their gene pool, bioresources and ecosystems. Thus, it is necessary to have access to FAIR-enabled data to better manage their bioresources and balanced socio-economic development. However, making FAIR-enabled data available is daunting for many researchers and organisations due to the lack of awareness, efficient data management tools, infrastructure and skills.

The BEXIS2 system has been developed considering the researchers' data management skills and requirements from the biodiversity and ecology community. The system is modular and extensible and it aims to support researchers, data managers and developers in data activities through the research data lifecycle while ensuring reproducibility. The unique approach of self-assessing the BEXIS2 features to grade its conformance with the FAIR indicators reveals the system is well suited to support researchers to meet the challenge of making available FAIR-enabled data. Although the BEXIS2 system has been developed, based on data management requirements predominantly from the biodiversity and ecology domain, the generic design allows the scientific scope of use beyond these domains.

### Funding

The development of BEXIS2 has been funded by the German Science Foundation (DFG) as part of the BExIS++ project (189571761). Funding for specific extensions and modules is further provided by the DFG through the projects: Biodiversity Exploratories, AquaDiva, iDiv and GFBio (193925721, 218627073, 202548816, 229241684) and BMBF-funded Managing Multimedia Data for Science (MAMUDS) project (01DH16009).

## Web location (URIs)

Download page: https://github.com/BEXIS2

Mailing list: bexis-support@uni-jena.de

## Technical specification

Platform: ASP.NET MVC

Programming language: C#

Operational system: Microsoft Windows Server/10, MS IIS

Service endpoint: NHibernate,PostgreSQL

## Repository

Type: GitHub

Browse URI: https://github.com/BEXIS2

## Usage licence

### Usage licence

Other

### IP rights notes

GNU Lesser General Public Licence version 3.0 (LGPL) https://www.gnu.org/licenses/lgpl-3.0.en.html

## Additional information

BEXIS2 demo website- https://demo.bexis2.uni-jena.de/

BEXIS2 Tech Talk- https://www.youtube.com/channel/UC_yQqVdDxYoygk6snNcuAmw

## Figures and Tables

**Table 1.** Self-assessed scoring of BEXIS2 system’s features in conformance with the **Findability** principle of the RDA maturity model. [**E**], [**I**] and [**U**] represent Essential, Important and Useful priorities, respectively. In the Availability column, **D** and **C** stand for Default and Configurable by the system, respectively.

**FAIR Indicator**	**BEXIS2 Feature**	**Score**	**Availability**
RDA-F1-01M: Metadata is identified by a persistent identifier [E]	BEXIS2 is interoperable with DataCite (FABRICA) application and thus capable of generating, registering and assigning DOIs to the datasets.	4	C
RDA-F1-01D: Data is identified by a persistent identifier [E]	BEXIS2 is interoperable with DataCite (FABRICA) application and thus capable of generating, registering, minting and assigning DOIs to the datasets.	4	C
RDA-F1-02M: Metadata is identified by a globally unique identifier [E]	Every dataset is assigned a unique identifier, which is used to identify metadata as well as the data. Additionally, every version of a dataset has its own identifier.	4	D
RDA-F1-02D: Data is identified by a globally unique identifier [E]	Every dataset is assigned a unique identifier, which is used to identify metadata, as well as the data. Additionally, every version of a dataset has its own identifier.	4	D
RDA-F2-01M: Rich metadata is provided to allow discovery [E]	A minimum set of metadata attributes must be maintained in the BEXIS2 system for each dataset. Additionally, data managers are able to ingest into the system and activate larger and/or customised metadata schemas, for example, EML, Darwin Core, amongst others.	4	C
RDA-F3-01M: Metadata includes the identifier for the data [E]	The metadata and data are packaged as a dataset (Fig. 2) and share the same identifier. In the case of datasets stored outside of the system, a record is maintained within the metadata that is accessible to the data managers and data owners.	3	D
RDA-F4-01M: Metadata is offered in such a way that it can be harvested and indexed [E]	Metadata can be harvested through the APIs or the specific interface that follows the OAI-PMH Standard. The emitted metadata includes the schema, which allows harvesters to index it. The implementation of schema.org recommendations is in progress.	4	D

**Table 2.** Self-assessed scoring of BEXIS2 system’s features in conformance with the **Accessibility** principle of the RDA maturity model. [**E**], [**I**] and [**U**] represent Essential, Important and Useful priorities, respectively. In the Availability column, **D** and **C** stand for Default and Configurable by the system, respectively.

**FAIR Indicator**	**BEXIS2 Feature**	**Score**	**Availability**
RDA-A1-01M: Metadata contain information to enable the user to get access to the data [I]	The information about the access policy and data licences can be provided via the metadata schema. Providing this information is the responsibility of the data manager.	4	C
RDA-A1-02M: Metadata can be accessed manually (i.e. with human intervention) [E]	Metadata, primary data and their schemas can be created, updated and retrieved through the user interface (GUI), as well as the API.	4	D
RDA-A1-02D: Data can be accessed manually (i.e. with human intervention) [E]	Metadata, primary data and their schema can be created, updated and retrieved through the user interface (GUI), as well as the API.	4	D
RDA-A1-03M: Metadata identifier resolves to a metadata record [E]	Identifiers and DOIs resolve to the landing page of the corresponding dataset, which provides access to metadata and data.	4	D
RDA-A1-03D: Data identifier resolves to a digital object [E]	Identifiers and DOIs resolve to the landing page of the corresponding dataset, which provides access to metadata, data and schemas. Direct access to the data is also provided through both the GUI and the API.	4	D
RDA-A1-04M: Metadata is accessed through standardised protocol [E]	Metadata can be accessed via HTTP or OAI-PMH protocols in HTML or XML serialisation formats.	4	D
RDA-A1-04D: Data is accessible through standardised protocol [E]	Data and its schema can be accessed via HTTP or OAI-PMH protocols in HTML, TEXT or JSON serialisation formats.	4	D
RDA-A1-05D: Data can be accessed automatically (i.e. by a computer program) [I]	Metadata, primary data and their schemas can be accessed via REST API or OAI-PMH interface.	4	D
RDA-A1.1-01M: Metadata is accessible through a free access protocol [E]	Metadata, primary data and data schema can be accessed via HTTP or OAI-PMH protocols in HTML or XML serialisation formats, all open and free. By default, all metadata are openly accessible.	4	D
RDA-A1.1-01D: Data is accessible through a free access protocol [I]	Data and its schema can be accessed via HTTP or OAI-PMH protocols in HTML, TEXT or JSON serialisation formats, all open and free.	4	D
RDA-A1.2-02D: Data is accessible through an access protocol that supports authentication and authorisation [U]	Data are accessed via either the GUI or the APIs. Both utilise HTTP, which supports authentication and authorisation. Data access is controlled by fine-grained permissions.	4	D
RDA-A2-01M: Metadata is guaranteed to remain available after data is no longer available [E]	The system allows for creating datasets with metadata only in the first place. Deleting data elements does not affect the life of the metadata.	4	D

**Table 3.** Self-assessed scoring of the BEXIS2 system’s features in conformance with the **Interoperability** principle of the RDA maturity model. [**E**], [**I**] and [**U**] represent Essential, Important and Useful priorities, respectively. In the Availability column, **D** and **C** stand for Default and Configurable by the system, respectively.

**FAIR Indicator**	**BEXIS2 Feature**	**Score**	**Availability**
RDA-I1-01M: Metadata uses knowledge representation expressed in standardised format [I]	Predefined vocabularies can be incorporated. There is a limited possibility to link terminology terms to the metadata attributes. This is the responsibility of the individual instance.	3	C
RDA-I1-01D: Data uses knowledge representation expressed in standardised format [I]	Users are able to design data structures by specifying their variables, units and data types and re-use them for any dataset. Annotating the units and variable properties to appropriate ontologies is under implementation.	3	C
RDA-I1-02M: Metadata uses machine-understandable knowledge representation [I]	Standard metadata schemas, such as EML, can be installed and used, which provide a moderate degree of machine understandability. The adoption of schema.org is in progress. It is also feasible to map metadata elements to their equivalents in, for example, Dublin Core. However, in the current implementation, metadata are exported in XML. Support for JSON is under development. Annotation of schema elements with ontology terms is under consideration.	3	C
RDA-I1-02D: Data uses machine-understandable knowledge representation [I]	Data are shipped with its schema that defines the variables, their unit of measurement and validation rules. The implementation of semantic data annotation is done as a contributing project. Integration of the module into BEXIS2 is under planning.	3	C
RDA-I2-01M: Metadata uses FAIR-compliant vocabularies [I]	The system integration with well-known terminology servers, such as the GFBio Terminology Server, which act as the data source for the designated metadata attributes is planned.	2	C
RDA-I2-01D: Data uses FAIR-compliant vocabularies [U]	In tabular data, variables can be defined according to international standards (e.g. SI units)	2	C
RDA-I3-01M: Metadata includes references to other metadata [I]	Datasets can have qualified relations to other datasets in the system. Metadata contain links to other internal self-defined entities (e.g. publication). Currently, these references are not part of the metadata as default, but as a part of the dataset package during download. Datasets maintain their version tracking; however, by default, the latest version is served. Linking to other internal datasets inside metadata is under implementation. Metadata may contain references to other metadata depending on the setup of the metadata schema. This property is customer-specific.	3	C
RDA-I3-01D: Data includes references to other data [U]	There are qualified references such as data versioning and related internal or external data inside the system. However, this is not served alongside the data. Data may contain references to other data depending on the setup of the data structure. This property is customer-specific.	2	C
RDA-I3-02M: Metadata includes references to other data [U]	Datasets can be linked to other datasets, as well as other internal resources. They are always linked to their previous versions. Metadata may also contain references to any other external resource (e.g. using URL). Exposing such links in the metadata is under implementation.	3	D
RDA-I3-02D: Data includes qualified references to other data [U]	There are qualified references, such as data versioning and related internal or external data inside the system. However, this is not served alongside the data. Data may contain references to other data depending on the set-up of the data structure. This property is customer-specific	2	C
RDA-I3-03M: Metadata includes qualified references to other metadata [I]	Datasets can have qualified relations to other datasets in the system. Metadata contain links to other internal self-defined entities (e.g. publication). Datasets maintain their version tracking; however, by default, the latest version is served. Linking to other internal datasets inside metadata is also possible. Metadata may contain references to other metadata depending on the set-up of the metadata schema. This customer-specific property is under implementation.	3	C
RDA-I3-04M: Metadata include qualified references to other data [U]	Metadata can have qualified links to other datasets, as well as other internal resources. They are always linked to their previous versions. Metadata may also contain references to any other external resource (e.g. using URL). Exposing links to the previous versions of the dataset, other datasets, as well as and other internal resources in the metadata is under implementation.	3	C

**Table 4.** Self-assessed scoring of BEXIS2 system’s features in conformance with the **Reusability** principle of the RDA maturity model. [**E**], [**I**] and [**U**] represent Essential, Important and Useful priorities, respectively. In the Availability column, **D** and **C** stand for Default and Configurable by the system, respectively.

**FAIR Indicator**	**BEXIS2 Feature**	**Score**	**Availability**
RDA-R1-01M: Plurality of accurate and relevant attributes are provided to allow Reuse [E]	The system has the capacity to generate rich metadata. Metadata can provide information about the data, contributors, geo-temporal extent, context, schema, software used, licensing, versioning and identification of the designated datasets. The system allows the use of existing metadata schemas, also in co-existence.	4	C
RDA-R1.1-01M: Metadata includes information about the licence under which the data can be reused [E]	BEXIS2 provides site and tenant-level terms and conditions policies. In addition, each individual dataset can have its own licence.	4	D
RDA-R1.1-02M: Metadata refers to a standard reuse licence [I]	BEXIS2 provides site and tenant-level terms and conditions policies. In addition, each individual dataset can have its own licence. BEXIS2 is able to restrict the list of licences available to its users to choose from.	4	C
RDA-R1.1-03M: Metadata refers to a machine-understandable reuse licence [I]	The list of licences can be obtained and chosen from a standard vocabulary, such as SWO (https://www.ebi.ac.uk/ols/ontologies/swo)	4	C
RDA-R1.2-01M: Metadata includes provenance information according to community-specific standards [I]	Each and every change to the metadata generates a new version in the system. Therefore, the complete change history is maintained and accessible. However, this provenance data are not yet communicated via a community-specific standard. At the moment, BEXIS2 maintains a linear forward versioning scheme similar to that of source control systems.	2	D
RDA-R1.2-02M: Metadata includes provenance information according to a cross-community language [U]	Each and every change to the metadata commits a new version to the system. Therefore, the complete change history is maintained and accessible. However, this provenance data are not yet communicated via a language, such as PROV-O.	1	D
RDA-R1.3-01M: Metadata complies with a community standard [E]	BEXIS2 is able to ingest multiple communities and/or cross-community metadata standards for use. BEXIS2 is shipped with some community standard metadata schemas (e.g. EML, Dublin Core).	4	C
RDA-R1.3-01D: Data complies with a community standard [E]	BEXIS2 provides tabular data in open formats such as TXT, CSV, TSV, as well as public (community applied) formats, such as EXCEL. BEXIS2 maintains the original file format that is used by the data owner and/or the community. For tabular data, users are able to design data structures by specifying its variables, units and data types and re-use them for any dataset. Thus, users are able to implement community standards.	4	C
RDA-R1.3-02M: Metadata is expressed in compliance with a machine-understandable community standard [E]	BEXIS2 is able to ingest multiple communities and/or cross-community metadata standards for use. BEXIS2 is shipped with some community standard metadata schemas (e.g. EML, Dublin Core). Metadata are accessible via API.	4	C
RDA-R1.3-02D: Data is expressed in compliance with a machine-understandable community standard [I]	For tabular data, users are able to design data structures by specifying its variables, units and data types and re-use them for any dataset. Thus, users are able to implement community standards. However, data are not yet provided in a machine-understandable way.	2	C
